# Stroke Recurrence among Stroke Patients Referred for Driving Assessment and Rehabilitation: A Cohort Study

**DOI:** 10.3390/jcdd10020083

**Published:** 2023-02-16

**Authors:** Narayanaswamy Venketasubramanian, Mei Leng Chan

**Affiliations:** 1 Raffles Neuroscience Centre, Raffles Hospital, Singapore 188770, Singapore; 2 Department of Occupational Therapy, Tan Tock Seng Hospital, Singapore 308433, Singapore

**Keywords:** stroke, recurrence, driving

## Abstract

Returning to driving is one of the priorities for stroke survivors. However, the fear of the risk of recurrent stroke has led to concern about allowing driving post-stroke. This study was performed to study the impact of various vascular risk factors on stroke recurrence among drivers referred to our national referral center for Driving Assessment and Rehabilitation Program (DARP). Medical records of subjects who were diagnosed to have a stroke and were referred to DARP were retrospectively reviewed. Data on demographics (age and gender) and vascular risk factors (hypertension—HT, diabetes mellitus—DM, hyperlipidemia—HL, cigarette smoking—SM, previous stroke—PS, and heart disease—HD) were collected. Subjects were contacted and records scrutinized for a report of recurrent stroke. A total of 133 subjects were recruited, median 54 years (range 20–77 years), 95.5% male, 59.4% had HT, 32.3% DM, 65.4% HL, 43.6% SM, 3.8% PS, and 8.3% HD. Over a median follow-up of 30 months (range 1–78 months), the recurrence rate of stroke was 11.3%, 3.69/100 patient-years. On uni-variable analysis, the risk of stroke recurrence rose with age (HR 1.08, 95%CI 1.02–1.15, *p* = 0.01) and heart disease (HR 5.77, 95%CI 1.46–22.83, *p* = 0.01). On multivariable analysis, only age remained significant (HR 1.07, 95%CI 1.00–1.13, *p* = 0.045). Among those aged > 60 years, the HR was 3.88 (95%CI 1.35–11.20, *p* = 0.012). The risk of stroke recurrence is higher among older drivers and is not influenced by other vascular factors.

## 1. Introduction

Stroke is a major cause of death and disability globally, and even more so in the Asia Pacific region [1]. Based on the Global Burden of Disease study in 2019, incident cases of stroke comprised 12.2 million (95% UI 11.0–13.6) prevalent cases of stroke comprised 101 million (93.2–111), and deaths from stroke comprised 6.55 million (6.00–7.02), with 143 million (133–153) disability-adjusted life year lost (DALYs) due to stroke [2]. Globally, stroke was the second-leading cause of death, comprising 11。6% [10.8–12.2] of total deaths and the third-leading cause of death and disability combined (5.7% [5.1–6.2] of total DALYs). The total number of strokes and deaths due to stroke has been increasing—this trend is expected to continue with the growth in the global population and increased longevity, and especially in low-income countries and those in economic transition where an increase in the incidence and prevalence of vascular risk factors, often poorly controlled, is expected [3]. 

In addition to causing mortality and morbidity, there are other complications that may arise after stroke, both in the acute phase and subsequently [4]. These include infections (e.g., pneumonia and urinary tract infection), deep venous thrombosis, pulmonary embolism, malnutrition, decubitus ulcers, incontinence, pain, fatigue, apathy, insomnia, depression and cognitive impairment; some of these are more likely among severely disabled stroke survivors. Another potential complication of stroke is stroke recurrence. A recent meta-analysis involving 1,075,014 stroke patients in 37 studies reported the risk of recurrent stroke as 7.7% at 3 months, 9.5% at 6 months, 10.4% at 1 year, 16.1% at 2 years, 16.7% at 3 years, 14.8% at 5 years, 12.9% at 10 years, and 39.7% at 12 years after the initial stroke [5]. 

Persisting disability is a challenge many stroke survivors face. In a long-term cohort study of 139 stroke patients, at 4 years after stroke 42.3% still had limitation of their activities (Barthel Index < 95), and 28.2% had restriction of participation (Frenchay Activities Index FAI < 30) [6]. In a longer cohort study of 349 stroke patients at 6 years post-stroke, 65% had a lower level of activity compared to their status pre-stroke based on the FAI [7]. Among the principle aims of a rehabilitation program is for the patient to the regain functional independence and self-care for activities of daily living and, if possible, return to the pre-stroke functional state, which would include work and leisure [8].

Driving a car is an important instrumental activity of daily living that allows independence, mobility, freedom to be able to manage one’s affairs, and for some, a means to earn a living [9]. A person unable to return to driving may be negatively impacted in overall health due isolation from family, friends, community, and otherwise inaccessible resources. Among cohort studies, in a single-centre study in Australia (n = 359), 26.5% returned to driving within one month after stroke [10], while in a multi-centre study in Korea (n = 629), 66.1% had resumed driving in a mean of 2.15 months [11]. In a US study (n = 156), 31% returned to driving in six months [12], while in a longer Canadian multi-centre study (n = 290), 61% returned to driving after one year [13].

There have been concerns about the safety of driving after stroke—a sudden severe clinical event occurring while driving after a previous stroke may lead to serious accidents [14]. This would include the occurrence of a recurrent stroke. However, a meta-analysis of 12 articles failed to show a robust increased risk of motor vehicular collision (MVC) after stroke—an association was found in one out of three case-controlled studies (OR 1.9, 95%CI 1.0–3.9), and one of five cohort studies (RR 2.71, 95%CI 1.11–6.61); two out of four cross-sectional studies using computerized driving simulators did find a more than two-fold risk of MVCs among those with stroke compared with controls—one of the studies only included those with middle cerebral artery stroke [15]. The uncertainty about safety has led to varying recommendations for post-stroke driving [14]. An expert panel has recommended driving cessation for one year after a TIA or stroke, and return to driving a commercial motor vehicle be allowed only after successful completion of a comprehensive neurological evaluation, neuropsychological assessments, and on-road testing [16]. This study was thus performed to investigate stroke recurrence and its risk factors among drivers referred to our national Driving Assessment and Rehabilitation Program (DARP) after their stroke.

## 2. Materials and Methods

This study was performed in Tan Tock Seng Hospital, Singapore, a tertiary level hospital with the country’s oldest and largest Department of Rehabilitation Medicine. It is the site of our national DARP, which is run by trained occupational therapists [17]. The evaluation includes an off-road assessment of physical, cognitive, and visual abilities needed for driving, and then an on-road assessment with a certified driving instructor. A report is then prepared for the referring doctor to assist in the certification for fitness to drive.

In this retrospective cohort study, potential subjects were identified by reviewing the DARP registration records for all those who were attended to with a medical diagnosis of stroke. The medical case records of these subjects were then reviewed. Data on demographics (age, gender, and ethnicity) and recorded history of vascular risk factors (hypertension, diabetes mellitus, hyperlipidaemia, cigarette smoking, previous stroke, or heart disease) were collected.

Hypertension was defined as a documented diagnosis of hypertension or on blood pressure-lowering medications. Diabetes mellitus was defined as a documented diagnosis of diabetes mellitus or on glucose-lowering medications. Hyperlipidaemia was defined as a documented diagnosis of hyperlipidaemia or on lipid-lowering medications. Cigarette smoking was defined as ever having smoked. Previous stroke was defined as a documented diagnosis of previous stroke prior to the one the patient was referred for. Heart disease was defined as a documented diagnosis of heart disease including angina, myocardial infarction, coronary artery disease, or cardiac revascularisation.

Subjects were contacted and asked if they had suffered another stroke (primary study outcome) since their DARP assessment and when it had occurred. This was verified by review of their medical records. Stroke was defined as the sudden onset of a focal or global neurological deficit lasting 24 h or more due to a vascular cause [2].

Based on an estimated event rate of 10% [5], margin of error of 5% yielding a range 5–15%, and with an alpha of 0.05, the estimated sample size was 139 subjects. 

Data was expressed as means and SDs for normally distributed continuous variables, medians for non-normally distributed continuous variables, and proportions for categorical variables. Cox proportional hazards model was used to determine risk factors that significantly impacted on time to recurrent stroke. Data was analysed using STATA. The study was approved by the Ethics Committee.

## 3. Results

A total of 133 subjects were recruited, mean age 54 years, almost all male, with stroke vascular risk factors as described in Table 1. Based on the medical records, they were well at baseline, had minimal disability (modified Rankin score 0–1), prescribed evidence-based treatments, and cognitively intact. They were followed up for a median of 30 months. The frequency of stroke recurrence was 11.3%, 3.69/100 patient years. None were involved in a motor vehicular accident due to the recurrence.

Compared to those without recurrence, those with recurrence were older (*p* = 0.01) and had a higher frequency of heart disease (*p* = 0.01) (Table 2). There was no significant difference in gender or other vascular risk factors.

On multivariable analysis, only age remained significantly associated with stroke recurrence, HR 1.07 (95%CI 1.00–1.13, *p* = 0.045) (Table 3).

**Figure 1:**
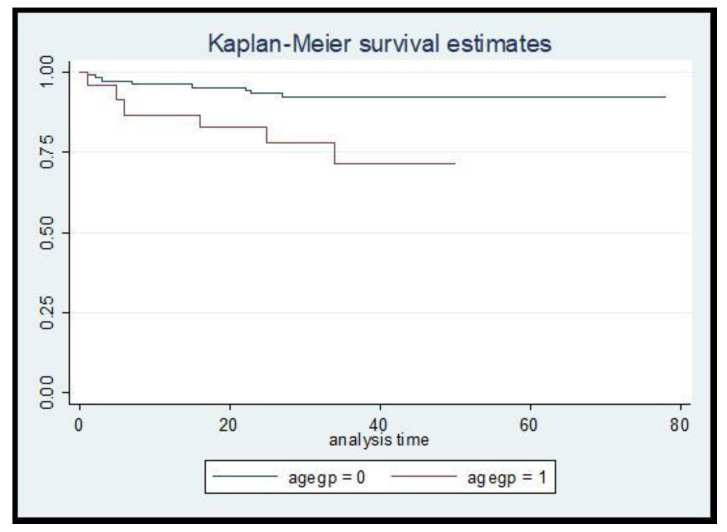
Survival curve for stroke recurrence among age ≤ 60 years vs. >60 years.

Compared to patients whose age was < 60 years old, those aged > 60 years old had a 3.88-fold risk of recurrence (95%CI 1.35–11.20, *p* = 0.012) (Figure 1). 

## 4. Discussion

Our study showed that among patients referred to a driving assessment rehabilitation program after their stroke, the risk of recurrent stroke over a median of 30 months is 11.3%, 3.69/100 patient years, with the main risk factor for recurrence being increasing age. The risk was almost 4-fold higher among those aged more than 60 years.

Cognitive impairment post-stroke can impair judgement while driving, including maintaining lane discipline performing complex driving tasks (e.g., making U-turns or left-turns) and regulating speed and braking time (16% longer in the stroke group, attributed to deficits in selective attention and motor accuracy) [16,18,19,20,21]. There is a range of cognitive assessment tools for evaluation post-stroke (most useful being the Stroke Driver Screening Assessment, Trail Making A and B tests, the Rey-Osterreith Complex Figure Design, and the Useful Field of View Test), aided by off-road simulators and on-road assessments [22,23]. Most physical impairments post-stoke can be overcome by suitable driving aids, for example, a spinner knob to improve steering and left-foot accelerator in an automatic vehicle to compensate for weakness and lack of coordination in the right leg. However, the other major concern is of ‘a second catastrophic event’ [14], the most notable being another stroke. The risk of a second stroke after the first has been reported in the meta-analysis to be 10.4% at 1 year [5]. Our patients had a recurrence rate of 11.3% at 30 months, 3.69/100 patient years, which is less than the reported rate. 

A number of scores have been developed and validated to predict the long-term risk of recurrence after the first stroke. These include the Essen Stroke Risk Score, Stroke Prognostic Instrument, Hankey score, and the Life Long After Cerebral ischemia score [24]. In all of these and other population-based studies, the various well-known risk factors are identified, such as hypertension, diabetes mellitus, hyperlipidaemia, cigarette smoking, other vascular disease, previous vascular events, and advancing age [25,26]. Our study is consistent with these earlier studies in finding age as a predictor of stroke recurrence, with a higher risk as age increases and a particularly high risk at an age above 60 years.

There are a number of efficacious treatments to reduce the risk of recurrent stroke [27,28]. These include antiplatelets, anticoagulants, carotid endarterectomy or stenting, left atrial appendage obliteration, aneurysm coiling or clipping, and arteriovenous malformation embolization and/or excision and/or focused irradiation. Highly subsidised healthcare, including these treatments, is available and readily accessible throughout Singapore. Patients are referred to DARP by their treating physicians and would likely have been prescribed the recommended treatments. Thus, it is probable that all subjects would be on evidence-based treatments including antithrombotics and other treatments to reduce their vascular risk factors. This may at least in part explain the low recurrence rate among our stroke patients.

Returning to driving is one of the priorities of stroke survivors, including for work and community reintegration, due to the impact on livelihood for commercial vehicle drivers, access to a convenient mode of transportation, and maintaining the quality of life for ordinary drivers. Studies have demonstrated varying levels of advice and evaluation to return to driving post-stroke. There are some published recommendations on fitness to drive after stroke, including country-specific guidelines from France, Nordic countries, Singapore, and the United States [29,30,31,32,33]. They have generally used the available evidence and considered issues peculiar and local to each country. While cognitive and physical abilities are important, the ability to safely drive a vehicle as determined by a formal driving assessment needs to be balanced against the risk of a catastrophic event, such as a second stroke [14]. To the latter end, physicians should ensure that their patients have been prescribed and are compliant with evidence-based medical therapies to reduce the risk of recurrence.

There are safety concerns when older individuals drive, as their ability to control a vehicle may be affected by cognitive sensory and physical factors, posing a danger to other road users [34]. As for cognitive and vehicle handling ability, an individualised functional assessment by a driver rehabilitation specialist, usually a certified driver-assessor occupational therapist, working with(out) a driving instructor, can competently evaluate driving ability and prescribe new devices followed by relevant on-road training practices that will help the patient to return to driving safely [35,36,37]. Thus, a close collaboration between the patient, physician, and driver rehabilitation specialist is key.

Our study has a number of clinical implications. The most important is the finding of a low risk of recurrence of stroke in the study population. The recurrence rate of 11.3% at 30 months, 3.69/100 patient years, is far less than the reported rate of 10.4% at one year in the recent meta-analysis. This suggests that in a country like Singapore with widely available, easily accessible, and highly subsidized healthcare, including for stroke, it is possible to provide evidence-based interventions to reduce the risk of recurrent vascular events to low levels. The lack of any MVC in the study further reduces the concern of lack of safety of driving after stroke. The provision of and compliance with treatment will need a close cooperation between the patient, family, and the treating physician. The other implication is the need for close cooperation between the treating physician and the DARP to carefully evaluate the cognitive and physical abilities of the stroke survivor to safely manage a motor vehicle. This was achieved by the comprehensive off-road and on-road assessments of driving competence by trained occupational therapists and certified driving instructors.

There are some limitations to this study. This is a single-centre retrospective study with a small number of subjects. Data was not collected on obesity or level of exercise; smoking history was based on self-report. Compliance to treatment and the adequacy of control of risk factors was not evaluated. However, this study was based on patients from our national referral centre for driving assessment, the follow-up time is substantial, and subject contact and medical record reviews were performed by the research team. While the study sample size of 133 was less than the estimated sample size of 139, the study still had 90% power at an alpha of 0.05 and beta of 0.1.

## 5. Conclusions

In conclusion, this study shows that the risk of stroke recurrence is highest among older drivers and appears not to be influenced by the presence of other vascular factors. The results of this study should be replicated as the findings may have implications on the certification of drivers as fit to drive after a stroke. We hope this paper will further support efforts to certify and allow enabled drivers to return to driving after their stroke.

## Figures and Tables

**Table 1:** Characteristics of recruited subjects at baseline.

Characteristic	
Number of subjects	133
Age (years)—median Range	54 20–77
Male (%)	95.5
Hypertension (%)	59.4
Diabetes mellitus (%)	32.3
Hyperlipidaemia (%)	65.4
Smoking (%)	43.6
Previous stroke (%)	3.8
Heart disease (%)	8.3
Follow-up (mo)—median Range	30 1–78
Stroke recurrence (%) (rate/100 patient years)	11.3 3.69

**Table 2:** Subject risk factors and stroke recurrence.

Risk Factor	Recurrent Stroke		*p*
	No N = 118	Yes N = 15	
Age (years)	52.14 (9.6)	59.50 (10.9)	0.01
Male	113	14	0.67
Hypertension	72	7	0.29
Diabetes mellitus	40	3	0.29
Hyperlipidaemia	79	8	0.30
Smoking	51	7	0.80
Previous stroke	4	1	0.54
Heart disease	7	4	0.01

**Table 3:** Regression analysis of subject risk factors and stroke recurrence.

Risk Factor		Univariable			Multivariable	
	Odds ratio	95%CI	*p*-value	Odds ratio	95%CI	*p*-value
Age (years)	1.08	1.02–1.15	0.01	1.07	1.00–1.13	0.045
Male	0.62	0.07–5.69	0.67			
Hypertension	0.56	0.19–1.64	0.29			
Diabetes mellitus	0.49	0.13–1.83	0.29			
Hyperlipidaemia	0.56	0.19–1.67	0.30			
Smoking	1.14	0.39–3.38	0.80			
Previous stroke	2.04	0.21–19.52	0.54			
Heart disease	5.77	1.46–22.83	0.01	0.28	0.06–1.25	0.095

## Data Availability

The data presented in this study are available on request from the study site—Tan Tock Seng Hospital.
